# Prognostic significance of tumor-infiltrating lymphocytes in predicting outcome of distal cholangiocarcinoma in Thailand

**DOI:** 10.3389/fonc.2022.1004220

**Published:** 2022-12-13

**Authors:** Piyapharom Intarawichian, Sirada Sangpaibool, Piya Prajumwongs, Prakasit Sa-Ngiamwibool, Sakkarn Sangkhamanon, Waritta Kunprom, Malinee Thanee, Watcharin Loilome, Narong Khuntikeo, Attapol Titapun, Apiwat Jareanrat, Vasin Thanasukarn, Tharatip Srisuk, Vor Luvira, Kulyada Eurboonyanun, Julaluck Promsorn, Supinda Koonmee, Aileen Wee, Chaiwat Aphivatanasiri

**Affiliations:** ^1^ Cholangiocarcinoma Screening and Care Program (CASCAP), Khon Kaen University, Khon Kaen, Thailand; ^2^ Cholangiocarcinoma Research Institute, Khon Kaen University, Khon Kaen, Thailand; ^3^ Department of Pathology, Faculty of Medicine, Khon Kaen University, Khon Kaen, Thailand; ^4^ Department of Biochemistry, Faculty of Medicine, Khon Kaen University, Khon Kaen, Thailand; ^5^ Department of Surgery, Faculty of Medicine, Khon Kaen University, Khon Kaen, Thailand; ^6^ Department of Radiology, Faculty of Medicine, Khon Kaen University, Khon Kaen, Thailand; ^7^ Department of Pathology, Yong Loo Lin School of Medicine, National University of Singapore, National University Hospital, Singapore, Singapore

**Keywords:** tumor-infiltrating lymphocytes (TILs), distal cholangiocarcinoma, prognosis, growth pattern, predicting outcome

## Abstract

Patients with distal cholangiocarcinoma (dCCA) generally have poor outcomes because of late presentation and diagnosis. Therefore, prognostic factors for predicting outcomes are essential to improve therapeutic strategies and quality of life. Tumor-infiltrating lymphocytes (TILs) have been reported as a prognostic predictor in several cancers. However, their role in dCCA is still unclear. This study aimed to evaluate the association of TILs with outcome in patients with dCCA. Fifty-two patients were evaluated for the percentage rate of TILs in their cancers, and a median TIL level was used to divide the patients into two groups. Survival, multivariate, and correlation analyses were performed to determine the prognostic factors. Results showed that a low TIL level was associated with poor survival. Multivariate analysis revealed TILs as an independent factor for poor outcome. Moreover, TILs were markedly correlated with growth patterns, and both were applied to classify patients with dCCA. Subgroups of TILs with growth pattern incorporation improved stratification performance in separating good from poor patient outcomes. This study suggested that TILs could be a prognostic factor for predicting survival and for clustering patients with dCCA to improve prognostication capability. This finding may be incorporated into a new staging system for stratifying dCCA in Thailand.

## Introduction

The incidence of cholangiocarcinoma (CCA), a cancer of bile duct epithelium, has been reported globally to be the highest in the Northeastern region of Thailand, especially in Khon Kaen province (1, 2). The liver fluke, *Opisthorchis viverrini*, has been shown to be a major risk factor associated with the high incidence rate in this region, inducing carcinogenesis through several possible mechanisms (2–4). In general, CCA has high mortality rates because of the difficulty in attaining early diagnosis with patients often appearing with advanced stage/metastatic disease. Therefore, an accurate stratification and staging is important to enable better strategies for effective prognosis and treatment.

On the basis of anatomical localization, CCA is classified into three types comprising intrahepatic (iCCA), perihilar)pCCA), and distal CCA (dCCA) (5). Although they have similarities, there are some significant inter- and intratumoral differences that can influence the pathogenesis and outcome. Although rare in our region, this study focuses on dCCA because of the dismal outcome and the lack of capability of current staging systems to accurately classify and stratify patients after curative-intended surgery for optimum management (6, 7). Numerous reports have documented that dCCA appears more frequently in Western countries and North America, accounting for approximately 30% of all CCAs (8, 9). In contrast, the incidence rate is low in Southeast Asia; however, about 10% of all CCAs occur in Thailand (6, 7) with approximately 8% in our cohort (10). Most patients with dCCA have poor outcomes (late-stage and short survival time) due to advanced disease at presentation with lymph node and distant metastasis (11). Surgery is usually the first choice for palliative treatment, whereas chemotherapy and radiotherapy are the secondary options (12). The 5-year survival time and rate are approximately 17–20 months and 10%–25%, respectively (7–10, 13, 14). There is, therefore, an urgent need to improve the clustering of patients with dCCA for precise prognostication and management.

Prognostic predictors are essential factors for predicting the outcome of patients with cancer. Although the American Joint Committee on Cancer and The Union for International Cancer Control (AJCC/UICC) staging system is the most widely used for cancer staging, several studies still debate the suboptimal performance in their cancer cohorts, especially for CCA. The study of prognostic factors is needed to improve the prediction of outcomes in patients with CCA. In addition, prognostic factors are also incorporated into the staging system to improve the performance of stratification. Some studies have demonstrated, particularly in dCCA, that the AJCC/UICC staging system is not satisfactory to classify patients, resulting in ambiguities in each staging. Hence, several prognostic factors, such as growth pattern (10), histological grade (15, 16), and cancer markers (17), have been applied to predict the outcome of patients with CCA or improve the staging system for accurate classification.

Tumor-infiltrating lymphocytes (TILs) consist of T cells, B cells, and NK cells. They are the primary immune cells that can infiltrate against pathogens or cancer cells. TILs are a manifestation of the host immune response against pathogens or cancer cells. Numerous reports have already suggested the potential role of TILs as a prognostic factor for various cancers, such as colorectal (18), lung cancer (19), breast (20), and liver cancers. Current reports suggest that TILs can be used as a significant predictor for the survival and outcome of many solid tumors (18–22). Moreover, it can relate to the highest likelihood of response to therapy (23, 24). However, the utility of TILs as a prognostic prediction of dCCA outcome is still unclear.

This study, therefore, aimed to investigate the relationship between TIL levels and the outcome of patients with dCCA. The assessment of TILs on hematoxylin and eosin (H&E)– stained histological sections was evaluated according to the International TILs Working Group (ITWG) guideline. The correlation between TIL level and clinicopathological characteristics was explored.

## Materials and methods

### Patients

Patients diagnosed with dCCA between 2004 and 2016 at the Srinagarind Hospital, Faculty of Medicine, Khon Kaen University, Thailand, were studied. Exclusion criteria included patients with small biopsies and those who survived less than 30 days after surgery with probable perioperative causes of death. A total of 52 patients with curative-intended surgery was finally included. The follow-up time was at least 5 years. This study was approved by the Ethics Committee for Human Research, Khon Kaen University (HE641613).

### Recorded data

Intraoperative data collection included sex, age, tumor size, growth patterns, surgical margin, and characteristics of surrounding organs. The specimens were examined with relevant tissue blocks taken by a pathologist for routine tissue processing. Formalin-fixed paraffin-embedded tissue blocks were sectioned at 5 μm (25) and stained with H&E. The 2019 WHO classification criteria were adopted for pathological diagnosis (26). By light microscopy, the following histomorphological data were recorded: growth patterns, histological type, histological grade, surgical margin, lymphovascular invasion, and lymph node metastasis. Evidence for distant metastasis was retrieved from the medical records. Finally, the gross examination and pathological findings were correlated according to the eighth AJCC staging manual (27).

### Tumor-infiltrating lymphocyte evaluation

**Figure 1:**
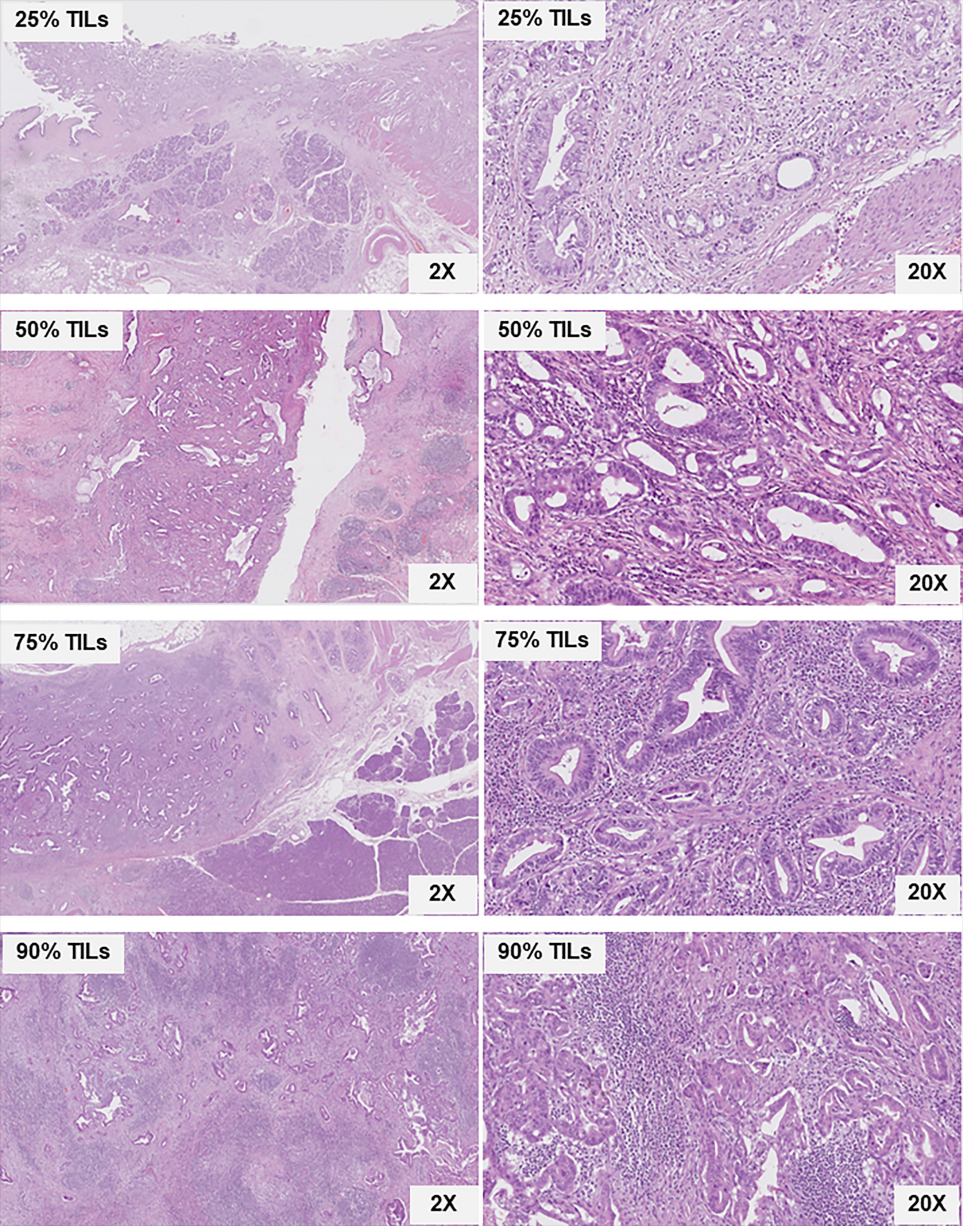
Histological sections of various distal cholangiocarcinoma stained with H&E showing tumor glands, stroma, and TILs. The percentage rates of TILs distribution in the stroma and tumor areas comprising 25%, 50%, 75%, and 90%, according to the ITWG guideline, are shown at ×2 and ×20 magnification.

TILs were evaluated on H&E sections from surgically resected specimens. According to the ITWG guideline (28), average percentage rates of TILs in each case were identified and evaluated as TIL levels (**Figure 1**). The guideline recommended assessing the average TIL percentage rates from the stromal area that was filled with mononuclear cells around the tumor border and in the tumor area. The TIL score included all mononuclear cells (i.e., lymphocytes and plasma cells) but excluded polymorphonuclear leukocytes . The average TIL percentage rates in each case are calculated from stromal TIL and intratumoral TIL percentage rates. The denominator used to determine the percentage rate of stromal TILs was the area of stromal tissue (i.e., the area occupied by mononuclear inflammatory cells over the total stromal area), and, similarly, for intratumoral TILs, the tumor cell area was the denominator.

### Growth pattern estimation

The growth pattern estimation criteria were applied according to a previous report by Kunprom et al. (10). The resection specimens were trimmed and photographed with the tumor growth pattern/s recorded at the time of grossing, followed by subsequent histological confirmation. The growth patterns comprised intraductal (ID), periductal infiltrating (PI), and mass-forming (MF) patterns. The patterns were estimated in increments of 10% to establish the proportion of each pattern (ID, PI, or MF) or combinations of patterns (ID + PI, ID + MF, PI + MF, or ID + PI + MF).

### Pathological diagnosis

There were four major histological types: papillary adenocarcinoma, tubular adenocarcinoma, papillotubular adenocarcinoma, and adenocarcinoma (Not otherwise specified, NOS). Papillary, tubular, and papillotubular adenocarcinomas were classified into well or moderately differentiated cancers (2019 WHO classification) (26). Adenocarcinoma, NOS, was defined as poorly differentiated bile duct cancer, lacking well-formed papillary or tubular structures.

### Statistical analysis

Only patients with complete datasets were included in the statistical analyses. Statistics for categorical data were performed with the χ2-test (or the Fisher’s exact test, as appropriate). For the survival rate and median survival time from the date of surgery for dCCA until death from dCCA, the Kaplan–Meier model was used, which is applicable for survival analyses; the log-rank test was used to compare the difference in survival. Perioperative causes of death were excluded from this analysis. Multivariate analysis was performed using the Cox regression model to determine the prognostic factors. For the percentage rate of growth pattern decision criteria, a 20% growth pattern estimation cutoff value was used as this showed significantly different overall survival (OS) between each type of growth pattern (29). All statistical analyses were performed using SPSS version 23. *P*-values of less than 0.05 were considered to be statistically significant.

## Results

### Estimation of TIL levels in patients with distal cholangiocarcinoma

**Figure 2:**
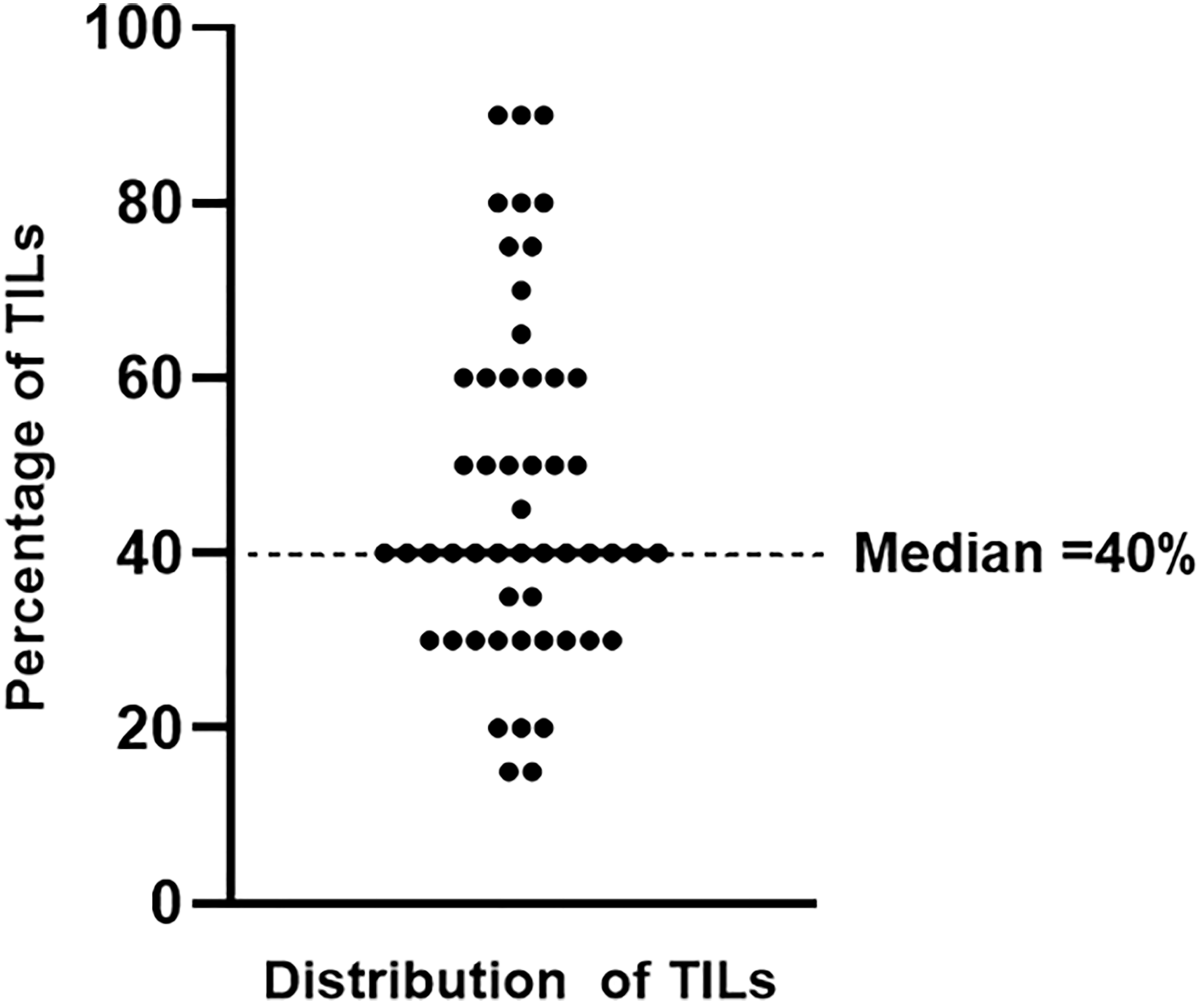
The distribution of TIL levels in distal cholangiocarcinoma. Patients with dCCA were divided in low and high TIL levels according to the median value.

According to the ITWG guideline, the percentage rate of TILs was evaluated by two consensus pathologists. Subsequently, patients with dCCA were separated into two groups using a median percentage rate of TILs. The median TIL level of 40% was used as a cutoff value for dividing the patients with dCCA: TIL level ≤ 40%, low level of TILs (n = 29); and TIL level > 40%, high level of TILs (n = 23) (**Figure 2**). On the basis of the two groups, the survival, correlation, and prognostic analyses were determined by the log-rank, chi-square (or Fisher’s exact test, as appropriate), and Cox regression tests, respectively.

### Baseline clinicopathological features

A total of 52 patients with dCCA who underwent curative-intended surgery were included in this study. The baseline clinicopathological features of this study are shown in **Supplementary Table 1**. Briefly, the median age was used to divide them into two groups: median age ≤ 59 (n = 25, 48%) and >59 (n = 27, 52%) years. There were male (n = 33, 66%) and female (n = 19, 34%) patients.

Gross examination revealed the median tumor size to be 2 cm (range, 0.2–24 cm). On the basis of tumor size, there were three groups: ≤2 cm (n = 16, 30.8%), >2 cm (n = 13, 25%), and unknown size (n = 23, 44.2%). The tumors were categorized according to growth patterns that were based on prognostic outcomes that have been previously reported. They comprised ID mixed types (ID + PI, ID + MF, and ID + PI + M) (n = 23, 44%) and without ID mixed types (PI, MF, and PI + MF) (n = 29, 56%) (10). The surgical margin was investigated microscopically to be free from tumor, R0 (n = 39, 75%), and involved by tumor, R1 (n = 13, 25%).

Pathological evaluation by three pathologists showed the distribution of histological types: papillary adenocarcinoma (P) (n = 17, 33%); tubular adenocarcinoma (T) (n = 26, 50%); papillotubular adenocarcinoma (P+T) (n = 7, 13%); and adenocarcinoma, NOS (n = 2, 4%). Histological grades comprised well (n = 49, 94%) and moderately/poorly (n = 3, 6%) differentiated carcinomas. Nuclear pleomorphism (grade) was based on morphology, size, shape, and variation of nuclei, as described in Materials and Methods, and comprised scores 1 (n = 4, 8%), 2 (n = 27, 52%), and 3 (n = 21, 40%). Nucleoli prominence, consisting of three groups, comprised absent nucleoli (n = 3, 6%), nucleoli present at ×10 magnification (n = 25, 46%), and nucleoli present only at ×40 magnification (n = 24, 48%).

According to eighth AJCC staging system, T categories comprised T1 (n = 6, 11%), T2 (n = 18, 35%), T3 (n = 14, 27%), and unknown T (n = 14, 27%). Lymph node metastasis (LN) following the eighth AJCC staging system was divided into two groups: LN0 (n = 29, 56%) and LN1 (n = 23, 44%). Distant metastasis (M) comprised M0 (n = 45, 87%) and M1 (n = 7, 13%). The metastatic sites included liver parenchyma (n = 2), hepatoduodenal tissue (n = 1), omentum (n = 2), and peritoneum (n = 2). Subsequently, TIL levels and all clinicopathological features were further analyzed through univariate analysis by the log-rank test and the prognostic risk factors or through multivariate analysis by Cox regression.

### The survival, univariate and multivariate analysis of TILs, and clinicopathological features in distal cholangiocarcinoma

The univariate and multivariate analyses of the possible risk factors for survival of patients with dCCA are shown in **Table 1**. The univariate analysis of survival showed that the growth patterns without ID components had a significantly shorter median OS than those with ID components (OS = 11 vs. 32 months, HR = 12.19, *p* < 0.001). The involved R1 status surgical margin had markedly inferior OS to the R0 status (OS = 12 vs. 24 months, HR = 3.67, *p* < 0.001). For T, LN, and M categories by the eighth AJCC staging system, the reference groups of each category—T1, LN0, and M0—had OS significantly better than T1 and T2, LN1, and M1, respectively. Interestingly, low TIL levels (reference group) had markedly shorter OS than high TIL levels (OS = 12 vs. 27 months, HR = 0.34, *p* = 0.001). The significant clinicopathological features in univariate analysis were further investigated in multivariate analysis. The results showed that the growth pattern, M category, and TILs were statistically significant in multivariate analysis (*p* < 0.001, 0.05, and 0.001, respectively). Therefore, this information suggested that the growth pattern, M category, and TILs were the independent factors for the prognostication of outcome in patients with dCCA. The association of growth pattern without ID components, M1 status, and low level of TILs had a higher risk for poor outcome than their references, approximately 16.43-, 6.45-, and 6.49-fold (when high TIL level was reference group), respectively (**Table 1**).

**Table 1:** Univariate and multivariate analysis of survival in patients with distal cholangiocarcinoma.

Feature	Univariate analysis				Multivariate analysis		
	N = 52	OS (month)	HR (95% CI)	*p*-value	HR (95% CI)	*p*-value
Age (year)						
≤59	25	17	1		–	–
>59	27	23	0.88 (0.50–1.55)	0.649	–	–
Gender						
Male	33	16	1		–	
Female	19	23	0.69 (0.37–1.25)	0.219	–	–
Tumor size (range, 0.2–24 cm)						
≤2 cm	16	16	1		–	
>2 cm	13	22	1.10 (0.51–2.42)	0.804	–	–
Unknown*	23	–	–	–	–	–
Growth pattern^#^						
With ID mixed type	23	32	1		1	
Without ID mixed type	29	11	12.19 (5.01–29.63)	**<0.001**	16.43 (4.27–63.19)	**<0.001**
Surgical margin (R)^$^						
R0	39	24	1		1	
R1	13	12	3.67 (1.78–7.56)	**<0.001**	1.36 (0.61–2.99)	0.451
Histological type						
Papillary carcinoma	17	15	1			
Tubular carcinoma	26	16	0.95 (0.50–1.81)	0.885	–	–
Papillotubular carcinoma	7	25	1.04 (0.42–2.58)	0.930	–	–
Adenocarcinoma (NOS)	2	–	–	–	–	–
Histological grade						
Well differentiated	49	22	–		–	–
Moderately/poorly differentiated	3^&^	–	–	–	–	–
Nuclear pleomorphism						
1	4^&^	–	–			
2	27	22	–	–	–	–
3	21	10	–	–	–	–
Nucleoli prominence						
Absent	3^&^	–	–			
Present (×1x magnification)	24	23	–	–	–	–
Present (×40 magnification)	25	16	–	–	–	–
T categories						
T1	6	32	1		1	
T2	18	15	3.57 (1.18–10.75)	**0.024**	2.12 (0.73–6.18)	0.168
T3	14	17	3.04 (0.99–9.33)	**0.052**	1.77 (0.58–5.35)	0.314
T4	–	–	–	–	–	–
Unknown	14	–	–	–	–	–
Lymph metastasis (LN)						
LN0	29	24	1		1	
LN1	23	12	2.09 (1.17–3.75)	**0.012**	1.26 (0.59–2.68)	0.551
Distal metastasis (M)						
M0	45	23	1		1	
M1	7	4	2.93 (1.28–6.71)	**0.011**	6.65 (1.04–42.76)	**0.046**
TILs level						
Low (TILs ≤40%)	29	12	2.96 (1.56–5.61)	**0.001**	6.49 (2.49–16.98)	**<0.001**
High (TILs >40%)	23	27	1		1	

*The data was not available in clinical report.

**^#^** ID mixed type comprised ID, ID + PI, ID + MF, and ID + PI + MF, whereas without ID mixed type comprised PI, MF, and PI + MF [intraductal (ID), periductal infiltrating (PI), and mass-forming (MF) patterns).

^$^The surgical margin was investigated microscopically to be free from tumor, R0, and involved by tumor, R1.

^&^N < 5 was excluded to estimate survival analysis.

OS, overall survival; HR, hazard ratio.

Bold values means statically significant value.

### Relation of TILs with clinicopathological features

The 52 dCCA cases were divided into two groups according to median TIL cutoff value: low (TILs < 40%, n = 29, 56%) and high (TILs ≥ 40%, n = 23, 44%) TIL levels (**Figure 2**). The χ2-test was performed to compare the correlation between TIL levels and clinicopathological variables. The results revealed that TIL levels showed statistically significant correlation with nuclear pleomorphism (*p* = 0.010), growth pattern (*p* = 0.031), and gender (*p* = 0.037). In other words, the clinicopathological features relating to poor survival, including marked nuclear pleomorphism (score 3) and growth pattern without ID components (PI, MF, and ID + PI + MF), were associated with low levels of TILs. In addition, there was a significant correlation between low levels of TILs with gender, especially male patients (**Table 2**). Remarkably, TILs and growth patterns, which were the independent factors in multivariate analysis, showed a significant correlation. Thus, we incorporated both factors to create subgroups for improving the prognostic prediction of the outcomes of patients with dCCA.

**Table 2:** Correlation between the TIL level and clinicopathological features in patients with distal cholangiocarcinoma.

Feature	TIL level			*p*-value
	Low (TILs ≤40%) n (%)	High (TILs >40%) n (%)	Total n (%)	
Age (year)				
≤59	13 (52%)	12 (48%)	25 (100%)	0.598
>59	16 (59.3%)	11 (40.7%)	27 (100%)
Gender				
Male	7 (36.8%)	12 (63.2%)	19 (100%)	0.037
Female	22 (66.7%)	11 (33.3%)	33 (100%)
Tumor size				
≤2	9 (56.3%)	7 (43.8%)	16 (100%)	0.340
>2	5 (38.5%)	8 (61.5%)	13 (100%)
Growth pattern (with or without ID component)				
ID mixed type	9 (39.1%)	14 (60.9%)	23 (100%)	0.031
Without ID mixed type	20 (69%)	9 (31%)	29 (100%)
Surgical margin				
R0	21 (53.8%)	18 (46.2%)	39 (100%)	0.629
R1	8 (61.5%)	5 (38.5%)	13 (100%)
Histological type				0.539
Papillary adenocarcinoma	10 (58.8%)	7 (41.2%)	17 (100%)
Tubular adenocarcinoma	14 (53.8%)	12 (46.2%)	26 (100%)
Papillotubular adenocarcinoma	3 (42.9%)	4 (51.1%)	7 (100%)
Adenocarcinoma, NOS	2 (100%)	0 (0%)	2 (100%)
Histological grade				
Well differentiated	27 (55.1%)	22 (44.9%)	49 (100%)	0.588
Moderately/poorly differentiated	2 (66.7%)	1 (33.3%)	3 (100%)
Eighth AJCC staging system				
T categories				
T1	3 (50%)	3 (50%)	6 (100%)	0.573
T2	11 (61.1%)	7 (38.9%)	18 (100%)
T3	6 (42.9%)	8 (57.1%)	14 (100%)
T4	–	–	–	
LN category				
LN0	15 (51.7%)	14 (48.3%)	29 (100%)	0.510
LN1	14 (60.9%)	9 (39.1%)	23 (100%)
M category				
M0	23 (51.1%)	22 (48.9%)	45 (100%)	0.086
M1	6 (85.7%)	1 (14.3%)	7 (100%)
Nuclear pleomorphism (score)				
1	2 (50%)	2 (50%)	4 (100%)	**0.010**
2	10 (37%)	17 (63%)	27 (100%)
3	17 (81%)	4 (19%)	21 (100%)
Nucleoli prominence (magnifications)				
Absent	1 (33.3%)	2 (66.7%)	3 (100%)	0.669
Present (×10)	15 (60%)	10 (40%)	25 (100%)
Present (×40)	13 (54.2%)	11 (45.8%)	24 (100%)

Bold values means statically significant value.

### Incorporation of TILs and growth patterns improved the prognosis performance of patients with distal cholangiocarcinoma

**Figure 3:**
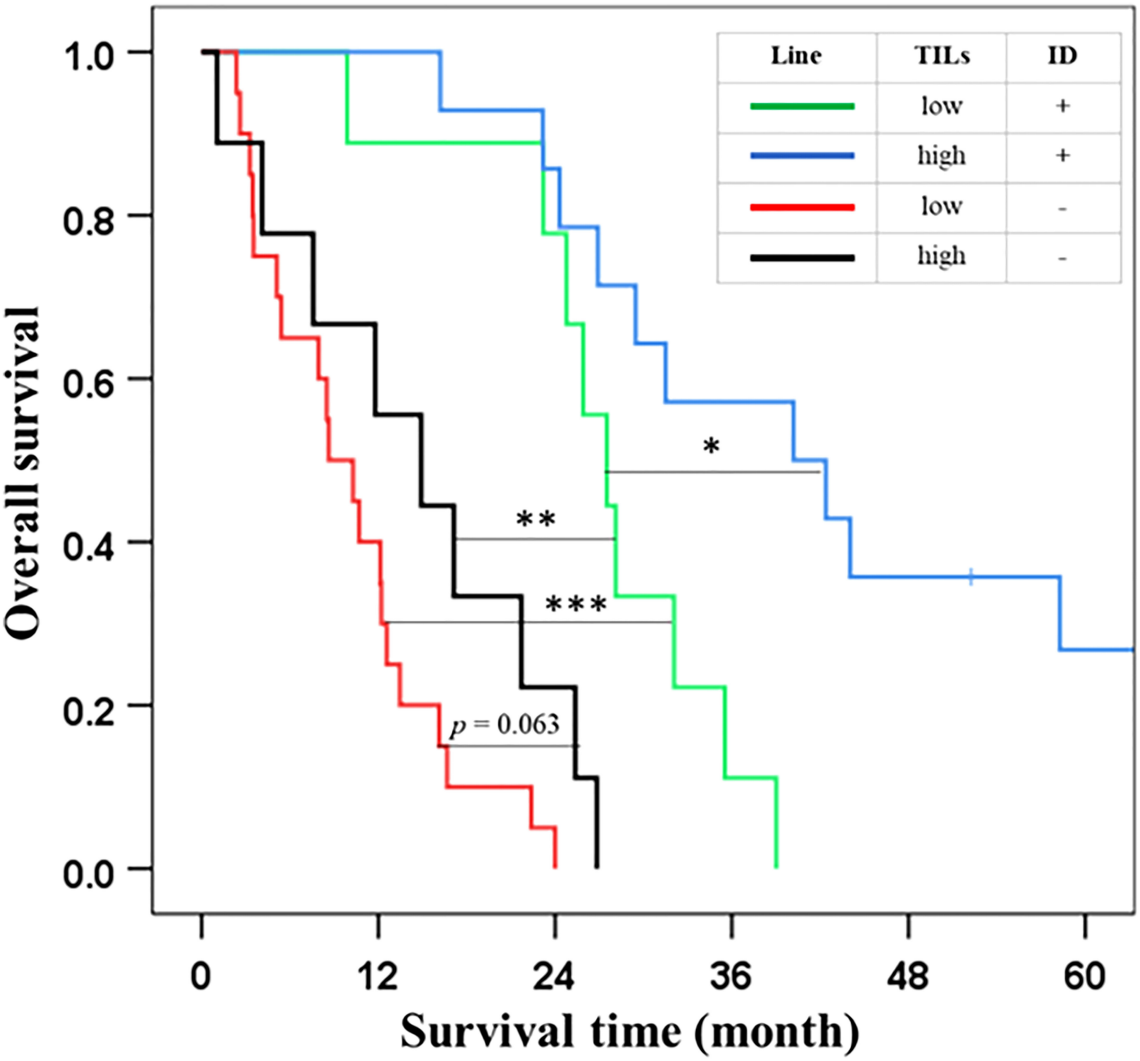
The outcome of patients with distal cholangiocarcinoma by subgroup analysis on incorporation TIL levels and growth pattern. Each line represents a combination of TIL levels and growth pattern appearance. The asterisks *, **, and *** indicate *p <*0.05, 0.01, and 0.001, respectively.

From multivariate analysis (**Table 1**) and correlation (**Table 2**), we found that the TIL levels and growth patterns significantly impacted on survival of patients with dCCA. In addition, both features revealed a significant correlation in the χ2-test. Therefore, we hypothesized that incorporating TILs and growth patterns might improve the prognostic prediction of the outcomes of patients with dCCA. In the experimental design, we divided the patients into four groups based on **Table 2**: (i) low TIL level + with ID components (n = 9), (ii) high TIL level + with ID components (n = 14), (iii) low TIL level + without ID components (n = 20), and (iv) high TIL level + without ID components (n = 9). The results showed that the outcome of patients with dCCA was extremely worsened when presented with low levels of TILs and without ID components (OS = 9 months). Then, the outcome of patients with high TIL level + without ID components and low TIL level + with ID components was 15 and 28 months, respectively. In contrast, high TIL level + with ID components had good prognostic outcomes (OS = 40 months) when compared to the other groups (**Table 3** and **Figure 3**). This information suggested that the incorporation of TIL levels and growth patterns improved the prognostic prediction of the outcomes of patients with dCCA.

**Table 3:** Subgroup analysis of the TIL levels and growth patterns in the outcome patients with distal cholangiocarcinoma.

Subgroups	N (%)	OS (month)	Univariate analysisHR (95% CI)	*p-*value
1. Low TILs level + with ID components	9	28	1	
2. High TILs level + with ID components	14	40	0.28 (0.097–0.80)	**0.018**
3. Low TILs level + without ID components	20	9	10.48 (3.60–30.53)	**<0.001**
4. High TILs level + without ID components	9	15	4.67 (1.60–13.60)	**0.005**

Bold values means statically significant value.

## Discussion

Distal CCA is one of three major types of CCAs. It has a higher incidence rate in Western countries and North America (8, 9, 13) when compared to Southeast Asia. However, the presence of *Opisthorchis viverrini* in Northeastern Thailand has contributed significantly to the high incidence rate, accounting for 10% of all global CCAs (6, 7). Of this, the incidence rate of dCCA in Thailand is relatively low. Almost all patients with dCCA present with advanced stages, such as with lymph node (stage III) and distant metastasis (stage IV) (11). Surgery is usually the first choice for palliative treatment, whereas chemotherapy and radiotherapy are secondary options (12). The 5-year survival time and rate are approximately 20 months and 25%, respectively (7–9, 13, 14). Thus, early diagnosis and precise prognosis are important for accurate clustering and treatment plans.

The AJCC/UICC staging system is a popular tool for clustering patients with cancer into stages based on the aggressive nature or expansion of cancers, especially CCA. Currently, the eighth edition of AJCC/UICC staging system is improved from the sixth and seventh editions. In dCCA classification in the eighth edition, there are two significant changes from the seventh edition: (i) T depth of invasion (DOI) separating T1, T2, and T3 (T1–T3) (27, 30); and (ii) addition of LN2 in which lymph node metastasis involves more than three nodes to separate LN0 and LN1 (27, 31). The overall performance of the eighth AJCC/UICC staging system in dCCA classification is better than the older editions (27, 30–33). Nevertheless, several reports debated that the eighth AJCC staging system is still unusable and shows low performance in several cohorts because T and LN categories are quite rigorously and stringently to perform DOI and positive lymph nodes as similar as the eighth AJCC staging system. In addition, some cohorts may have smaller number of patients leading to reduced stratification performance. Therefore, prognostic factors are proposed to replace or improve the eighth AJCC staging system (10, 17, 34–38). Bolm et al. investigated the correlation of cancer biomarkers, especially serum carbohydrate antigen 19-9 (CA19-9), with the survival of patients with dCCA. This study suggested that a high level of CA 19-9 correlated with regional lymph node metastases and shorter survival (17).

In addition, Ji et al. suggested that neutrophil-to-lymphocyte ratio can be used to predict poor survival of patients with dCCA (38). Recently, our previous publication proposed gross or growth patterns comprising ID, PI, and MF growth types that have been reported to be correlated with survival of patients with CCA (39, 40). According to the outcome of each growth pattern, we separated patients with dCCA into two groups, with or without ID components. The results revealed that patients with ID components (ID, ID + PI, ID + MF, and ID + PI + MF) have better survival than patients without ID components (PI, MF, and PI + MF). This study suggested that growth pattern acts as a prognostic factor for the survival of dCCA. In addition, growth pattern without ID components correlated with lymph node metastasis that is a strong negative impact prognostic factor in the outcomes of patients with dCCA (10).

Although growth patterns worked well to separate good and poor outcomes of dCCA, this study has some limitations, as some cases showed no correlation between ID components and good survival or without ID components and poor survival. Therefore, our study aimed to improve the prognostic factor by finding a combination factor. This study proposed incorporation of TILs.

**Figure 4:**
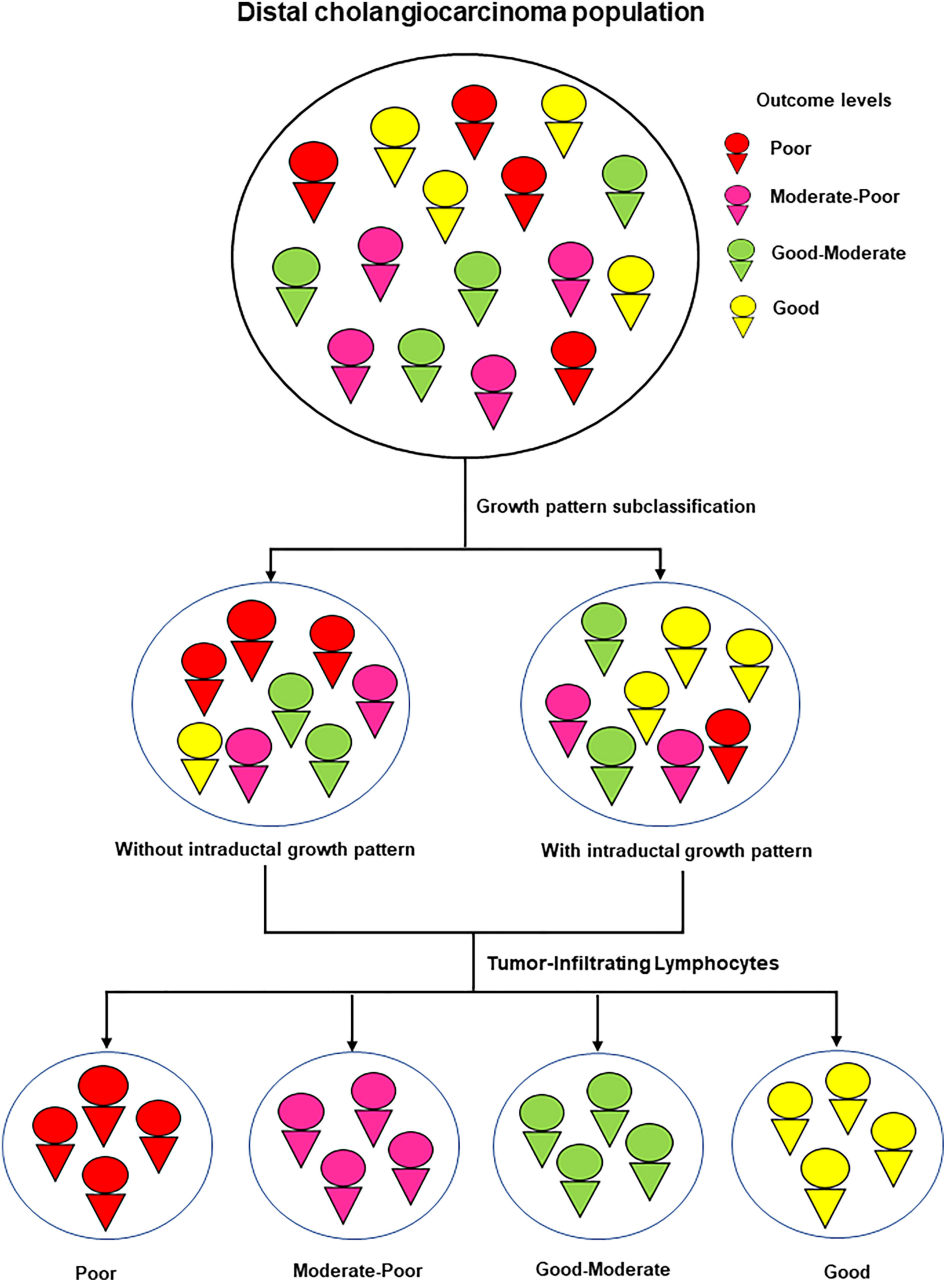
Schematic summary for the study.

TILs have been reported as a prognostic factor of survival in several cancers. Previous reports consistently recommended that the low levels of TILs correlated with poor survival of patients with cancer (18–22). The evidence for the role of TILs in cancer progression has been reported in several cancers (18–22, 41). The mechanism of action of TILs is associated with the host immune response to eliminate pathogens or cancers *via* adaptive immunity mediated by T and B lymphocytes that have been reported to have effective and sustained antitumor responses, to improve patient survival (42, 43) and response to therapy (44). Moreover, TILs have also been suggested as a predictor of response to neoadjuvant chemotherapy, leading to favorable clinical outcomes (41, 45, 46). Similarly, in CCA, we created the cut point to divide patients into two groups: low (TILs ≤ 40%) and high (TILs > 40%) levels. Our finding showed that a low level of TILs was associated with poor survival, whereas a high level of TILs correlated with better survival. Furthermore, TILs were also an independent factor in the prognosis outcome of patients with dCCA. Interestingly, the correlation analysis found that TILs correlated with growth patterns, and both were the independent factors in the multivariate analysis. Therefore, this study aimed to improve prognostic performance and accurate prediction. Subgroup analysis was performed based on the basis of four groups: low TIL level + with ID components, high TIL level + with ID components, low TIL level + without ID components, and high TIL level + without ID components (**Table 3**, **Figure 3**). The survival results of subgroup analysis showed that TILs could improve the stratification performance of growth pattern subclassification. There were four groups that were extracted from two groups of growth patterns. Previous research on the correlation of TILs and growth patterns has not been widely reported, and the explanation for the correlation is still unclear. Zhao et al. studied the correlation of tumor-infiltrating (PI) growth pattern and tumor immune environment in esophageal squamous cell carcinoma. Their findings suggested that PI was associated with tumor immune environment, especially the low level of TILs, lymph node metastasis, overall survival, the absence of tertiary lymphoid structures, deep tumor invasion, and poorly differentiated phenotype (47). This information revealed that a combination of PI and low levels of TILs was associated with the worst prognosis, such as the absence of tertiary lymphoid structures, deep tumor invasion, and poorly differentiated phenotype. In CCA, PI was classified to have the worst prognosis in growth patterns as MF or the combinations. Several studies have suggested that MF and PI (without ID components) correlated with the worst prognosis in patients with CCA: lymph node metastasis (10), poorly differentiated phenotype (48), tumor recurrence (49, 50), and poor survival (10). Similarly, in our study, growth patterns, MF, and PI (without ID components) are associated with a low level of TILs, which might represent expanded immunosuppression by the aggressive tumor subtypes (growth patterns, MF, and PI), resulting in the poor outcome of patients with dCCA. Therefore, the combination of the growth pattern and TILs is helpful for the risk stratification of prognosis (**Figure 4**).

This study revealed that TILs are significantly correlated with growth patterns and nuclear pleomorphism and are associated with the prognostic outcomes of patients with dCCA. In addition, combining TILs with growth patterns can improve the prognostic prediction of dCCA patient outcomes. This information may be helpful for further study in other types of CCA and other solid tumors.

## Data availability statement

The original contributions presented in the study are included in the article/**Supplementary Material**. Further inquiries can be directed to the corresponding author.

## Ethics statement

This study was approved by the Ethics Committee For Human Research, Khon Kaen University (HE641613). The patients/participants provided their written informed consent to participate in this study.

## Author contributions

Conceptualization: PI, SiS, and CA. Funding acquisition: CA. Sample collection and diagnosis: PI, SiS, PS-N, SK, SaS, PI, WK, NK, AT, AJ, VT, TS, VL, KE, WL, JP, and CA. Analysis and interpretation of data: CA and PP. Supervision: CA. Writing—original draft: PI, SiS, and PP. Writing—review and editing: PI, SiS, PP, WK, MT, AW, and CA. All authors approved the final version of the manuscript.
