# Supplementary material for: Prognostic significance of tumor-infiltrating lymphocytes in predicting outcome of distal cholangiocarcinoma in Thailand
Source: Front Oncol. 2022 Dec 13;12:1004220. doi: 10.3389/fonc.2022.1004220 (PMC9792867; doi:10.3389/fonc.2022.1004220)
Supplement: Supplementary file 1 [file Table_1.docx]

**Table**

**Supplementary Table 1.** Clinicopathological features of distal cholangiocarcinoma patients

| **Features** | **N = 52** | **%** |
| --- | --- | --- |
| Age (year) |  |  |
| ≤59 | 25 | 48 |
| >59 | 27 | 52 |
| Sex |  |  |
| Male | 33 | 66 |
| Female | 19 | 34 |
| Tumor size (range, 0.2-24 cm.) |  |  |
| ≤2 | 16 | 30.8 |
| >2 | 13 | 25 |
| Unknown^*^ | 23 | 44.2 |
| Growth pattern^**^ |  |  |
| ID mixed type | 23 | 44 |
| Without ID mixed type | 29 | 56 |
| Surgical margin (R)^***^ |  |  |
| R0 | 39 | 75 |
| R1 | 13 | 25 |
| Histological types |  |  |
| Papillary (P) | 17 | 33 |
| Tubular (T) | 26 | 50 |
| Papillotubular (P+T) | 7 | 13 |
| Adenocarcinoma, NOS | 2 | 4 |
| Histological grade |  |  |
| Well-differentiated | 49 | 94 |
| Moderately/ Poorly differentiated | 3 | 6 |
| Nuclear pleomorphism |  |  |
| 1 | 4 | 8 |
| 2 | 27 | 52 |
| 3 | 21 | 40 |
| Nucleoli prominence |  |  |
| Absent | 3 | 6 |
| Present (10x magnification) | 24 | 46 |
| Present (40x magnification) | 25 | 48 |
| T categories |  |  |
| T1 | 6 | 11 |
| T2 | 18 | 35 |
| T3 | 14 | 27 |
| T4 | - | - |
| Unknown* | 14 | 27 |
| Lymph node metastasis (LN) |  |  |
| LN0 | 29 | 56 |
| LN1 | 23 | 44 |
| Distant metastasis (M) |  |  |
| M0 | 45 | 87 |
| M1 | 7 | 13 |

***=** The data was not available in clinical report.

****=** ID mixed type composed ID, ID+PI, ID+MF and ID+PI+MF, while without ID mixed type composed PI, MF and PI+MF (Intraductal (ID), Periductal infiltrating (PI) and Mass-forming (MF) patterns.

*****=** The surgical margin was investigated microscopically to be free from tumor, R0 and involved by tumor, R1
